# Detection of virulence genes in Malaysian *Shigella *species by multiplex PCR assay

**DOI:** 10.1186/1471-2334-5-8

**Published:** 2005-02-14

**Authors:** Kwai Lin Thong, Susan Ling Ling Hoe, SD Puthucheary, Rohani Md Yasin

**Affiliations:** 1 Institute of Biological Sciences, Faculty of Science, University of Malaya, 50603 Kuala Lumpur, Malaysia; 2 Department of Medical Microbiology, Faculty of Medicine, University of Malaya, 50603 Kuala Lumpur, Malaysia; 3 Institute for Medical Research, Jalan Pahang, 50588 Kuala Lumpur, Malaysia

## Abstract

**Background:**

In Malaysia, *Shigella *spp. was reported to be the third commonest bacterial agent responsible for childhood diarrhoea. Currently, isolation of the bacterium and confirmation of the disease by microbiological and biochemical methods remain as the "gold standard". This study aimed to detect the prevalence of four *Shigella *virulence genes present concurrently, in randomly selected Malaysian strains via a rapid multiplex PCR (mPCR) assay.

**Methods:**

A mPCR assay was designed for the simultaneous detection of chromosomal- and plasmid-encoded virulence genes (*set1A*, *set1B*, *ial *and *ipaH*) in *Shigella *spp. One hundred and ten Malaysian strains (1997–2000) isolated from patients from various government hospitals were used. Reproducibility and sensitivity of the assay were also evaluated. Applicability of the mPCR in clinical settings was tested with spiked faeces following preincubation in brain heart infusion (BHI) broth.

**Results:**

The *ipaH *sequence was present in all the strains, while each of the *set1A*, *set1B *and *ial *gene was present in 40% of the strains tested. Reproducibility of the mPCR assay was 100% and none of the non-*Shigella *pathogens tested in this study were amplified. The mPCR could detect 100 colony-forming units (cfu) of shigellae per reaction mixture in spiked faeces following preincubation.

**Conclusions:**

The mPCR system is reproducible, sensitive and is able to identify pathogenic strains of shigellae irrespective of the locality of the virulence genes. It can be easily performed with a high throughput to give a presumptive identification of the causal pathogen.

## Background

Members of the genus *Shigella*, namely *S. flexneri*, *S. dysenteriae*, *S. sonnei *and *S. boydii *have caused and continue to be responsible for mortality and/or morbidity in high risk populations such as children under five years of age, senior citizens, toddlers in day-care centres, patients in custodial institutions, homosexual men and, war- and famine-engulfed people. Yearly episodes of shigellosis globally have been estimated to be 164.7 million and of these, 163.2 million were in developing countries and the remaining in industrialized nations. The mortality rate was approximately 0.7% [1]. A recent study by Lee & Puthucheary [2] on bacterial enteropathogens in childhood diarrhoea in a Malaysian urban hospital showed that *Shigella *spp. was the third most common bacteria isolated. *S. flexneri *and *S. dysenteriae *type 1 infections are usually characterized by frequent passage of small amounts of stool and mucus or blood. At times, watery stool followed by typical dysenteric stool maybe present with *S. dysenteriae *type 1 infection. *S. sonnei *and *S. boydii *infections are less severe with watery faeces but little mucus or blood.

Shigellosis is usually a self-limiting infection, however when it subsides, the intestinal ulcers heal with scar tissue formation. Uncomplicated recovery is usual and the organisms rarely cause other types of infections. Adversely, in 3 to 50% of cases, depending on the virulence of the strain, the nutritional and immune status of the host, the initial infection maybe followed by neurological complications or kidney failure. Serious complications do occur at greatest frequencies in malnourished infants, toddlers, older adults and immunocompromised individuals [3,4].

Virulence genes responsible for the pathogenesis of shigellosis may be located in the chromosome or on the *inv *plasmid borne by the organism. They are often multifactorial and coordinately regulated, and the genes tend to be clustered in the genome. Previously reported PCR-based detection methods concentrated mainly on the *ipaH *gene alone [5,6] or on *ipaH *and *ial *genes in two separate PCR assays [7,8]. As *ial *is found on the large *inv *plasmid which is prone to loss or deletions, this gene-based detection may give false negative results. *ipaH*, on the other hand, is present on both the *Shigella *chromosome and on a large plasmid and hence, it is a more stable gene to detect. However, the sole presence of *ipaH *is not an absolute indicator of virulence as loss or deletion of the plasmid renders the bacterium noninvasive and therefore, avirulent. *set1A *and *set1B *are chromosomal genes encoding *Shigella *enterotoxin 1 (ShET1), which cause the watery phase of diarrhoea in shigellosis [9,10]. *ial *and *ipaH *are responsible for directing epithelial cell penetration by the bacterium and for the modification of host response to infection, respectively [11-13].

Here, we describe the application of a multiplex PCR (mPCR) design for simultaneous detection of four virulence genes (*set1A*, *set1B*, *ial *and *ipaH*) in *Shigella *spp. and to determine the prevalence of these virulence genes in a random selection of Malaysian *Shigella *strains.

## Methods

### Bacterial strains and growth conditions

A total of 110 *Shigella *strains of *S. flexneri *(n = 84), *S. sonnei *(n = 15), *S. dysenteriae *(n = 10) and *S. boydii *(n = 1) were used in this study. These strains were isolated from patients with diarrhoea in Peninsular Malaysia from 1997–2000, and were provided by the Institute for Medical Research (IMR), Malaysia. Serotyping of the strains (*Shigella *antisera from Mast Diagnostics, UK) was carried out by the Bacteriological Unit, IMR. All the strains were checked on Salmonella-Shigella (SS) agar before being transferred to Luria Bertani (LB) agar plate, incubated overnight at 37°C for subsequent screening of virulence-associated genes. All strains were stored at -20°C in LB broth containing 15% glycerol.

### Development of mPCR

Boiled suspension of bacterial cells was used as DNA template. Previously described primers, obtained from Integrated DNA Techs, USA, for detection of the four virulence genes were applied to the template [8,14,15] (Table 1). Prior to combining all the four primer sets in an mPCR, each pair of primers was optimized singly in separate PCR assays. A typical 25-μl PCR reaction mixture for every primer set consisted of 1x PCR buffer B (Promega, USA), 4 mM MgCl_2_, 130 μM of each deoxynucleotide (dNTP), 0.5 μM of each primer, 1 U of *Taq *DNA polymerase (Promega, USA) and 2 μl of DNA template. Amplifications were carried out using a Robocycler Gradient 40 Temperature Cycler (Strategene Cloning Systems, USA). The cycling conditions were an initial denaturation at 95°C for 5 min, template denaturation at 95°C for 50 s, annealing at 55°C for 1.5 min, and extension at 72°C for 2 min for a total of 30 cycles, with a final extension at 72°C for 7 min.

**Table 1:** Primers used to identify various virulence-associated genes of *Shigella *spp.

**Primer**	**Virulence gene**	**Nucleotide sequences (5' → 3')**	**Size of amplicon (bp)**	**Reference**
ShET1A	*set1A*	TCA CGC TAC CAT CAA AGA TAT CCC CCT TTG GTG GTA	309	14
ShET1B	*set1B*	GTG AAC CTG CTG CCG ATA TC ATT TGT GGA TAA AAA TGA CG	147	14
ial	*ial*	CTG GAT GGT ATG GTG AGG GGA GGC CAA CAA TTA TTT CC	320	15
Shig1	*ipaH*	TGG AAA AAC TCA GTG CCT CT	423	8
Shig2		CCA GTC CGT AAA TTC ATT CT		

Based on the results of individual priming, an mPCR was designed. Various parameters such as concentrations of primers (0.5–0.8 μM), MgCl_2 _(2 to 4 μM), *Taq *DNA polymerase (0.6 to 4 U) and dNTPs (100–150 μM) and buffer strength (1.4X to 2.4X) were tested. The simultaneous gene amplifications were performed in a reaction volume of 25 μl consisting of 1.8X PCR buffer B (Promega, USA), 4 mM MgCl_2_, 130 μM of each dNTP, 0.3 μM of each ShET1B primer, Shig1 and Shig2 primers, 0.5 μM of each ShET1A and *ial* primers, 1 U of *Taq *DNA polymerase (Promega, USA) and 2 μl of DNA template. All the reaction mixtures were overlaid with 20 μl of sterile mineral oil. Amplifications were similarly carried out as above.

After initial screening, strain TH13/00 (*S. flexneri *2a) was chosen as a positive control for PCR assays. A negative control using sterile distilled water as template was included in every PCR assay. The DNA fragments were separated in 2% agarose gel.

### Reproducibility test

The mPCR assay was repeated at least twice with 28 strains to determine the reproducibility of the results, whereby the DNA template of a particular strain was freshly prepared for each repeat.

### Specificity test

The specificity of the mPCR assay was tested with 12 other non-*Shigella *pathogens: *Enterobacter cloacae*, *Salmonella *Paratyphi A (ATCC 9281), *S*. Paratyphi C, *S. *Typhimurium, *S. *Enteritidis, *S*. Typhi (ATCC 7251), *Listeria monocytogenes*, *Pseudomonas aeruginosa*, *Klebsiella pneumoniae*, *Citrobacter freundii*, *Escherichia coli *O157:H7 and *E. coli *O78:H11.

### Faecal spiking and sensitivity test

This was based on a modification of that described by Chiu and Ou [16]. Approximately 0.2 g of faeces from a healthy individual was suspended in 1 ml of brain heart infusion (BHI) (Oxoid Ltd., UK) and diluted 10-fold. Then, 1 ml of the diluted faecal suspension was inoculated into 4 ml of BHI and vortexed to obtain a homogenous mixture of broth-faecal suspension. Meanwhile, an overnight culture of *S. flexneri *2a was harvested and serially diluted 10-fold with BHI. Then, 250 μl of each dilution of cell culture was mixed with 250 μl of the broth-faecal suspension and 500 μl of BHI in a new eppendorf tube. The tubes were vortexed and preincubated at 37°C for 4 h without shaking. Simultaneously, 100 μl of each diluted culture was plated on LB agar (Oxoid Ltd., UK) to determine the number of viable bacteria in each dilution. After preincubation, mPCR assay was performed on the boiled lysates of each diluted culture. A pure culture of *S. flexneri *2a (TH13/00) and an unspiked faecal sample served as positive and negative controls.. The test was repeated with a spiked faecal sample of another healthy individual, and the average detection limit was reported.

### Screening of clinical specimens

0.2 g of each faecal sample from 10 patients suffering from diarrhoea in a local tertiary University Hospital was suspended in 1 ml of BHI and diluted 10-fold. A volume of 250 μl of broth-faecal suspension was inoculated into 5 ml of BHI and preincubated at 37°C for 4 h without shaking. Concurrently, 100 μl of the suspension was plated onto MacConkey and *SS *agar plates and incubated overnight at 37°C. mPCR assay was performed on the boiled lysate of the broth-faecal suspension after preincubation. A pure culture of strain TH13/00 and a *Shigella*-spiked faecal sample served as a positive control, whilst a PCR reaction mixture without bacterial DNA template and an unspiked faecal sample from a healthy individual acted as a negative control.

## Results

### Optimization strategies

**Figure 1:**
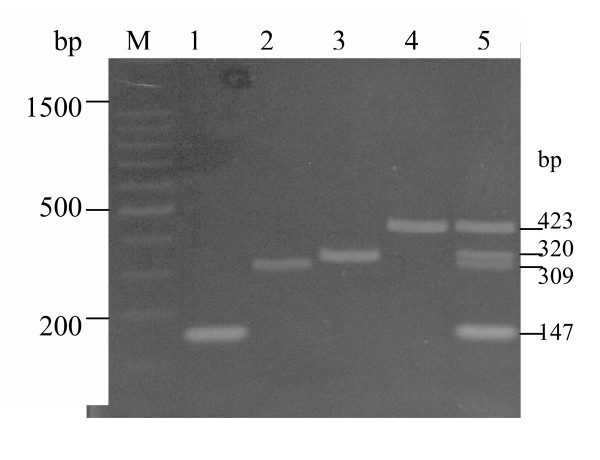
**Ethidium bromide-stained agarose gel showing PCR products. **Lane **M**, 100-bp DNA ladder (Promega); lane **1**, *set1B *gene product; lane **2**, *set1A *gene product; lane **3**, *ial *gene product; lane **4**, *ipaH *gene product; lane **5**, mPCR product.

A monoplex PCR for each primer set was initially carried out based on a published report [17]. Although the concentrations of MgCl_2 _(3 mM), dNTP (400 μM each) and primers (1 μM each) were used as recommended, unspecific bands were present together with intense primer-dimers. In order to reduce the background noise and primer-dimers, concentrations of 0.5 μM of each primer and 200 μM of each dNTP were used. Further optimizations of MgCl_2 _concentrations (2 to 4 μM) and dNTP (100,130 and 150 μM each) gave intense amplicons with a clean background in each monoplex amplification (Fig 1, lanes 1–4).

Initial attempts to amplify equally all the four genes in a single reaction using the reaction condition in monoplex PCR were not successful. A common practice in mPCRs involving any non-amplification of a required gene ('weak locus') is to increase the amount of primers of the gene at same time with a decrease of the amount of primers for all the loci that can be amplified, especially those with strong amplifications. Hence, the concentrations of primers for both *ipaH *(Shig) and *set1B *(ShET1B) were reduced to 0.3 μM each and the primers for both *set1A *(ShET1A) and *ial *(ial) genes were maintained at 0.5 μM each. Following optimization of the concentrations of Taq DNA polymerase (0.6 to 4U/25 μL), buffer strength (1.4 X to 2.4 X), dNTPs (140 to 220 μM) and annealing temperatures (49 to 59°C) (at a constant MgCl_2 _concentration of 4 mM), a more uniform amplification of all the genes with no background noise was obtained (Fig. 1 lane 5) at a final buffer concentration of 1.8X, 1 U *Taq *DNA polymerase, 130 μM dNTP each and annealing temperature of 55°C.

### Prevalence of virulence genes in the Malaysian strains

All the 110 strains of *Shigella *spp. tested showed the presence of *ipaH *(Table 2). Conversely, only 41% of the strains had both *set1A *and *set1B *genes, and *ial *gene. Almost all the *Shigella *strains tested positive for the tandem genes (87%) belonged to *S. flexneri *2a serotype. Among the predominant strains of *Shigella flexneri *in Malaysia, *ial *was found in serotypes 4a, 6, 3a, 2a, 1a and 3c. All the four genes were present only in *S. flexneri *2a and 3a.

**Table 2:** Prevalence of the four virulence-associated genes in Malaysian *Shigella *spp.

**Serotype**	**Total strains**	***set1B *(%)**	***set1A *(%)**	***ial *(%)**	***ipaH *(%)**
*S. flexneri *1a	3	0 (0)	0 (0)	1 (33)	3 (100)
1b	3	0 (0)	0 (0)	0 (0)	3 (100)
2a	47	41 (87)	41 (87)	19 (40)	47 (100)
3a	18	3 (17)	3 (17)	12 (67)	18 (100)
3c	10	0 (0)	0 (0)	1 (10)	10 (100)
4a	1	1 (100)	1 (100)	1 (100)	1 (100)
6	1	0 (0)	0 (0)	1 (100)	1 (100)
y	1	0 (0)	0 (0)	0 (0)	1 (100)
*S. sonnei*	15	0 (0)	0 (0)	2 (13)	15 (100)
*S. dysenteriae *2	10	0 (0)	0 (0)	8 (80)	10 (100)
*S. boydii *6	1	0 (0)	0 (0)	0 (0)	1 (100)

Total	110	45	45	45	110

### Reproducibility

Reproducibility for the detection of*set1A, set1B, ial *and *ipaH *genes assayed in the mPCR was 100%. None of the non-*Shigella *strains tested gave any amplification (data not shown).

### Sensitivity

The mPCR assay was tested on 10-fold dilutions of an overnight culture of *S. flexneri *2a. All the four virulence-associated genes were detected until 10^-3 ^dilutions (data not shown). This was equivalent to 2.45 × 10^5 ^lysate or a minimum of 490 cfu of shigellae per 25 -μLmPCR reaction.

### Faecal spiking and sensitivity

**Figure 2:**
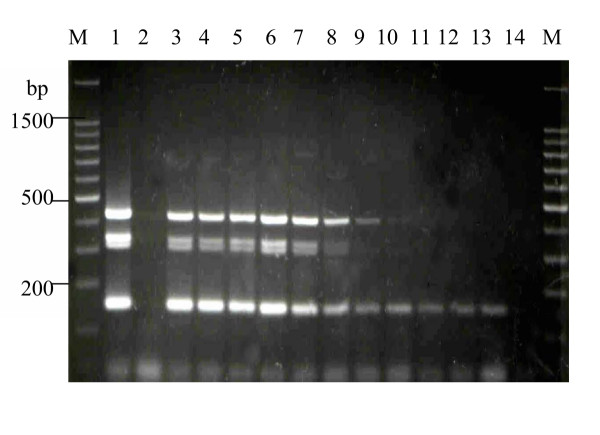
**Faecal-spiking and sensitivity result of mPCR. **Lane **M**, 100-bp DNA ladder (Promega, USA); lane **1**, TH13/00 (positive control); lane **2**, unspiked faeces (negative control); lane **3**, undiluted spiked faeces; lane **4**, 10^-1 ^dilution; lane **5**, 10^-2 ^dilution; lane **6**, 10^-3 ^dilution; lane **7**, 10^-4 ^dilution; lane **8**, 10^-5 ^dilution; lane **9**, 10^-6 ^dilution; lane **10**, 10^-7 ^dilution; lane **11**, 10^-8 ^dilution; lane **12**, 10^-9 ^dilution; lane **13**, 10^-10 ^dilution; lane **14**, "water blank".

An initial experiment using undiluted spiked faecal sample failed to give any PCR amplification (data not shown). When the faecal suspension was diluted and preincubated in BHI for 4 h, the mPCR assay was successful in detecting the presence of the four virulence genes at an average concentration of 5.0 × 10^4 ^colony-forming units (cfu) shigellae ml^-1 ^or approximately 100 cfu per reaction mixture (Fig. 2 lane 8).

### Clinical specimens

A preliminary study on the efficacy of the mPCR assay in the direct detection of the aforementioned *Shigella *virulence genes on faecal samples was tested on ten diarrhoeal patients. No mPCR product was detected although both the positive controls had amplifications. By conventional culture method, there was no growth of *Shigella *on the LB, MacConkey and *SS *agar plates.

## Discussion

Numerous studies had been performed to detect virulence genes in *Shigella *by monoplex PCRs [8,17,18]. Studies involving the combination of chromosomal- and plasmid-encoded virulence genes in a single assay for *Shigella *detection, on the other hand, are scarce. Although the optimization of mPCR is more tedious and difficult to achieve than monoplexes, the ease of screening a large number of specimens, once the system is optimized, far outweighs the initial problems. The present mPCR system encompasses the presence of virulence genes found in the *Shigella *chromosome and on the large *inv *plasmid. Hence, it can determine if the pathogenesis of a particular strain is attributable to its chromosome or the plasmid, or if the strain is still invasive or otherwise, in a single reaction.

Initially, the monoplex PCRs were carried out following reaction conditions as proposed by a previous report [17]. However, we could not reproduce their results and hence had to modify the PCR conditions. Our failure to reproduce identical results despite using similar reagent concentrations and amplification conditions maybe attributed to the different makes of PCR reagents and primers used. Broude *et al*. [19] had compared amplification efficiencies of two commercial *Taq *DNA polymerases and found that they displayed different specificity in PCR.

Preferential amplification of one target sequence over another is a known phenomenon in mPCRs and it is usually overcome by increasing the amount of primers for the weaker amplification simultaneously with a decrease of primer concentrations for the stronger amplification. Buffer concentration may also affect mPCR amplifications despite it being seldom considered during monoplex optimization works [20]. Upon adjustment of primers and buffer concentrations, specific and consistent amplification of all the genes in the multiplex combination was achieved.

Although other studies have demonstrated the presence of *ial *and *ipaH *in strains of enteroinvasive *Escherichia coli *(EIEC) [11,13,21], we had not applied the mPCR assay to EIEC strains. It is unfortunate that these strains were not available for our study as EIEC gives rise to similar illness as Shigellosis.

Our study supported the observations of Noriega *et al*. [9] and Vargas *et al*. [17] in local *Shigella *strains. Their studies showed that both *set1*A and *set1*B were present exclusively in *S. flexneri *2a. The complete correlation between the presence of both *set1A *and *set1B *showed that both genes are indeed found in tandem in the *Shigella *genome. In this study, almost all the *Shigella *strains positive for the presence of *set1A *and *set1B *(41/45 strains) belonged to *S. flexneri *2a, thus confirming previous works that both genes are highly conserved in this particular serotype [14].

Both the prevalence of *ial *and *ipaH *were independent of the four different species of *Shigella *tested. Though both *ial *and *ipaH *are responsible for invasion-related processes and are found on the *inv *plasmid, the *ial *gene cluster resides near a region of the plasmid, which is a hot spot for spontaneous deletions [22]. This probably explains the lower prevalence of *ial *(45/110 strains) than *ipaH *(110/110 strains) in the Malaysian *Shigella *strains. Since invasiveness is a prerequisite for virulence in shigellae and since most of these virulence genes are located on the large plasmid, these strains would have possessed the plasmid when first isolated from patients. Due to storage/subculturing, the plasmid might have been lost together with the virulence-associated genes. By virtue of multiple copies being present on both the chromosome and the *inv *plasmid [23], *ipaH *seemed to be less compromised by plasmid loss and/or deletions. As the sole presence of *ipa*H is not indicative of the invasive phenotype, our mPCR design, which incorporated three other virulence genes, could determine the invasiveness of *Shigella *strains in epidemiological studies.

Dilution of the faecal sample with BHI was performed to lower the levels of PCR inhibitors such as bilirubin, bile salts and heme in the faeces [16]. An additional step of preincubating the spiked faecal samples also helped to eliminate the natural inhibitors [24]. The short 4-h enrichment step would increase the total number of target sequences caused by more bacterial growth and the overall detection sensitivity of the assay. Although PCR cannot differentiate between dead and viable bacteria, enrichment helped to dilute the concentrations of dead bacteria, thus reducing the probability of detecting them by the subsequent mPCR assay. The sensitivity level achieved in the study was found to be comparable to other studies. Houng *et al*. [25] detected up to 7.4 × 10^4 ^cfu *shigellae *ml^-1 ^by amplifying the IS 630 sequences in *shigella *spp.. Yavzori *et al*. [24] reported a detection level of 10^4 ^cfu *shigellae *per gram of faeces with the use of *virF *primers. Although it has been reported that ingestion as low as 100 *shigellae *resulted in clinical disease [26], the highest percentage of volunteers having diarrhoea were administered doses of at least 10^4 ^viable organisms. Thus, the average detection limit of mPCR described in this study (5.0 × 10^4 ^cfu/ml) is within the common infectious dose for *shigellae*.

Results from the preliminary clinical screening were promising. Nevertheless, the consideration of other diarrhoeal pathogens being present in the clinical samples cannot be negated. More patient samples are warranted to thoroughly vet the robustness and applicability of the developed mPCR in clinical environments.

One limitation of the present mPCR system is its inability to differentiate *Shigella *spp., unlike the multiplex reactions based on specific *rfc *genes developed by Houng *et al*. [25]. For future research, either *set1A *or *set1B *may be omitted from the multiplex system as both genes are shown to exist tandemly. *rfc *primers of different *Shigella *origins maybe incorporated to enable the discrimination of *Shigella *spp. as well as the identification of virulent strains in one assay.

## Conclusions

We conclude that the mPCR system is able to identify pathogenic strains of shigellae irrespective of the locality of the virulence genes. The described assay is reproducible, sensitive, can be easily performed and is able to give a presumptive identification of the causal pathogen, which could be confirmed by culture techniques using selective media. An added advantage would be that EIEC, which gives a similar illness, might also be detected by this method, as EIEC also harbours *ial *and *ipaH *genes.

## Competing interests

The author(s) declare that they have no competing interests.

## Authors' contributions

SLLH carried out the experiments, data analysis and wrote the manuscript. RMY provided the bacterial strains. SDP contributed to the writing of the manuscript. TKL conceived and co-designed the study, provided input for writing and supervision of the study. All authors read and approved the final manuscript.

## Pre-publication history

The pre-publication history for this paper can be accessed here:


